# The Role of Epigenetic Regulation in Epstein-Barr Virus-Associated Gastric Cancer

**DOI:** 10.3390/ijms18081606

**Published:** 2017-07-25

**Authors:** Jun Nishikawa, Hisashi Iizasa, Hironori Yoshiyama, Munetaka Nakamura, Mari Saito, Sho Sasaki, Kanami Shimokuri, Masashi Yanagihara, Kouhei Sakai, Yutaka Suehiro, Takahiro Yamasaki, Isao Sakaida

**Affiliations:** 1 Department of Laboratory Science, Yamaguchi University Graduate School of Medicine, Ube, Yamaguchi 755-8505, Japan; g006up@yamaguchi-u.ac.jp (Ka.S.); m-yanagi@yamaguchi-u.ac.jp (M.Y.); 2 Department of Microbiology, Shimane University Faculty of Medicine, 89-1 Enyacho, Izumo City, Shimane 693-8501, Japan; iizasah@med.shimane-u.ac.jp (H.I.); yosiyama@med.shimane-u.ac.jp (H.Y.); 3 Department of Gastroenterology and Hepatology, Yamaguchi University Graduate School of Medicine, Ube, Yamaguchi 755-8505, Japan; munemune84@hotmail.com (M.N.); mari-s@zj8.so-net.ne.jp (M.S.); u003uj@yamaguchi-u.ac.jp (S.S.); sakaida@yamaguchi-u.ac.jp (I.S.); 4 Department of Oncology and Laboratory Medicine, Yamaguchi University Graduate School of Medicine, Ube, Yamaguchi 755-8505, Japan; sakaik@yamaguchi-u.ac.jp (Ko.S.); ysuehiro@yamaguchi-u.ac.jp (Y.S.); t.yama@yamaguchi-u.ac.jp (T.Y.)

**Keywords:** epigenetics, epstein-barr virus, gastric cancer, DNA methylation, microRNA, non-coding RNA, demethylating agent

## Abstract

The Epstein–Barr virus (EBV) is detected in about 10% of gastric carcinoma cases throughout the world. In EBV-associated gastric carcinoma (EBVaGC), all tumor cells harbor the clonal EBV genome. The expression of latent EBV genes is strictly regulated through the methylation of EBV DNA. The methylation of viral DNA regulates the type of EBV latency, and methylation of the tumor suppressor genes is a key abnormality in EBVaGC. The methylation frequencies of several tumor suppressor genes and cell adhesion molecules are significantly higher in EBVaGC than in control cases. EBV-derived microRNAs repress translation from viral and host mRNAs. EBV regulates the expression of non-coding RNA in gastric carcinoma. With regard to the clinical application of demethylating agents against EBVaGC, we investigated the effects of decitabine against the EBVaGC cell lines. Decitabine inhibited the cell growth of EBVaGC cells. The promoter regions of p73 and Runt-related transcription factor 3(RUNX3) were demethylated, and their expression was upregulated by the treatment. We review the role of epigenetic regulation in the development and maintenance of EBVaGC and discuss the therapeutic application of DNA demethylating agents for EBVaGC.

## 1. Introduction

Gastric cancer is the third leading cause of cancer-related mortality and is responsible for approximately 450,000 deaths worldwide each year [1,2]. Since gastric cancer has a heterogeneous etiology and histopathology [3], the optimum chemotherapy regimen for advanced cases—according to the molecular biological understanding of gastric cancer—has not been established.

**Figure 1:**
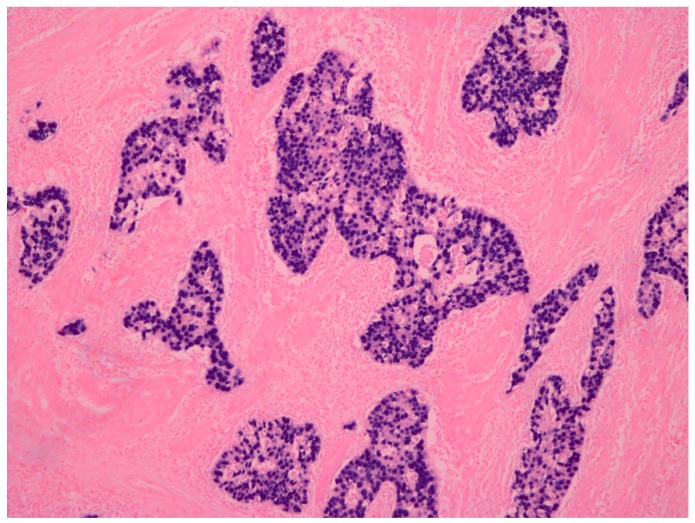
Representative case of Epstein-Barr virus (EBV)-associated gastric carcinoma. EBV-encoded small RNA 1 (EBER1) in situ hybridization (×100). Signals of EBER1 were detected in the nucleus of almost all cancer cells.

The Epstein–Barr virus (EBV) is a double-stranded DNA virus belonging to the herpes virus family, which latently infects B lymphocytes in the majority of adults. EBV is closely associated with both lymphoid and epithelial malignancies, such as Burkitt lymphoma and nasopharyngeal carcinoma [4]. The existence of the EBV genome in gastric cancer was first detected using a polymerase chain reaction in 1990 [5]. Since then, approximately 10% of gastric cancers in the world have been identified as positive for EBV [6]. In situ hybridization for EBV-encoded small RNA 1 (EBER1) has been used as the standard method for diagnosing EBV-positive gastric cancer. EBER1 signals can be detected in the nuclei of gastric cancer cells (Figure 1) [7,8]. Imai et al. [9] reported that EBV-associated gastric carcinoma (EBVaGC) resulted from the monoclonal proliferation of EBV-infected cells. This fact suggests that EBV plays an important role in the development of cancer [10].

Recently, the Cancer Genome Atlas (TCGA) of gastric adenocarcinomas [11] developed a novel classification system dividing gastric cancer into four molecular groups: (1) EBVaGC; (2) microsatellite instability (MSI); (3) chromosomal instability (CIN); and (4) genomically stable (GS) tumors. The characteristics of EBVaGC are reported to include the harboring of recurrent PIK3CA mutations, extreme DNA hypermethylation, and amplification of JAK2, PD-L1, and PD-L2. PIK3CA mutation and amplification of JAK2 could lead to PD-L1 overexpression [11]. Recently, many reports have addressed PD-L1 overexpression in EBVaGC [12,13,14]. It is possible that EBVaGC tumor cells evade immune reactions via the PD-1/PD-L1 immune check point pathway. Among these characteristics of EBVaGC, promoter hypermethylation and the downregulation of various tumor suppressor genes have already been reported [15,16,17,18,19].

In the present study, we review the role of DNA methylation in the development and maintenance of EBVaGC and discuss the therapeutic application of DNA demethylating agents for EBVaGC.

## 2. The Epigenetic Regulation of Latent Epstein–Barr Virus (EBV) Gene Expression

DNA methylation of the EBV genome has been studied intensively in EBV-associated tumors. It has been revealed that the expression of latent EBV genes is regulated by methylation in their promoter regions [20]. EBV-associated tumors exhibit three patterns of latency: types I, II, and III. Tumors with type I latency only express the EBV nuclear antigen (EBNA) 1 protein, as observed in Burkitt lymphoma. Tumors with type II latency express EBNA1, latent membrane protein (LMP) 1, and LMP2, as observed in EBV carrying T/NK cell lymphomas, nasopharyngeal carcinomas (NPC), and Hodgkin’s lymphoma. Tumors with type III latency express EBNA1-6 and LMP1 and LMP2s, as observed in immunoblastic lymphocytes arising in immunocompromised patients (Table 1). Similarly to Burkitt lymphoma, EBVaGCs are considered to show type I latency [9]. A limited set of viral genes, including EBNA1, EBERs, and Bam HI-A transcripts (BARTs), are expressed in neoplasms showing type I latency. EBNA promoters (Cp and Wp), which can transcribe all EBNAs, are hypermethylated in tumors showing type I latency, and the alternative EBNA promoter Qp is used [21,22]. These results indicate that the methylation status of the EBV genome regulates the pattern of latent gene expression in EBV-positive tumor cells.

There are reports that LMP2A mRNA was detected in some EBVaGCs [23,24]. EBV lytic genes, including BCLF1, BHRF1, BNLF2a, and BRLF1, were also expressed in EBVaGC [25]. Especially, BNLF2a is expressed in latently-infected tumor cells [26]. A systemic review by Ribeiro et al. showed that the most frequently expressed EBV latent proteins are EBNA1 (98.1%) and LMP2A (53.8%), whereas LMP1 and LMP2B are present in only 10% of cases. Lytic proteins, such as BARF0 and BARF1, and other lytic transcripts are present in almost half of the cases [27]. The pattern of latent EBV infection is more complex than the type I latency pattern (EBNA1, EBERs) initially described.

*Latent membrane protein 1* (*LMP1*) is a latent EBV gene that shows oncogenic activity and which is recognized and targeted by cytotoxic T lymphocytes. Approximately 60% of NPC cells carrying EBV express LMP1, while the frequency of LMP1 expressing cells in tumor tissue displays a high degree of variability. In these tumors, the expression of LMP1 is controlled by the methylation status of the LMP1 promoter (LMP1p). LMP1 is expressed in tumor cells that possess an EBV genome with an unmethylated LMP1p, not in tumor cells that possess an EBV genome with a highly methylated LMP1p [28]. The downregulation of LMP1 is also observed in EBVaGC. LMP1 promoter is hypermethylated in the EBV genome of EBVaGC tumors. DNA methylation is utilized as a host defense mechanism against viral DNA to suppress the expression of viral genes [29,30]. On the other hand, DNA methylation plays a crucial role in allowing EBV to escape from the surveillance of the host immune system.

The expression of latent EBV genes is strictly regulated through the methylation of EBV DNA. The methylation of viral DNA determines the type of EBV latency, which may contribute to the maintenance of EBVaGC.

## 3. DNA Hypermethylation in the Host Genome

The methylation of tumor suppressor genes is a key abnormality in EBVaGC. In the tumor cells of EBVaGC, CpG island methylation is frequently observed at promoters of various tumor-related genes, which plays important roles in the development and progression of gastric cancer [12,13]. The methylation frequencies of several tumor suppressor genes (i.e., APC, PTEN, and RASSF1A) and cell adhesion molecules (i.e., THBS1 and E-cadherin) are significantly higher in EBVaGC compared to EBV-negative controls [31,32,33]. Hypermethylation of somatostatin receptor 1, interferon regulatory factor 5, REC8 meiotic recombination protein, SLIT1, SLIT2, and SLIT3 was recently reported, and the methylation frequency was significantly higher in EBVaGC than in EBV-negative gastric cancer [34,35,36].

We compared the methylation status between EBVaGC and EBV-negative gastric cancer in patients matched for age, sex, histology, depth of invasion, and tumor stage. The methylation frequency in 12 of 16 tumor-related genes was significantly higher in EBVaGC than in EBV-negative gastric cancer by methylation-specific PCR (MSP). The methylation frequencies at six specific loci (MINT2, MINT31, p14, p16, p73, and Runt-related transcription factor 3(RUNX3)) in EBVaGC were significantly higher in comparison to EBV-negative gastric cancer [19]. The DNA methylation status was also examined in SNU-719 cells, which were derived from EBV-positive gastric adenocarcinoma, by the method of methylated CpG island recovery in a chip assay. We demonstrated that the promoter regions of p73, BLU, FSD1, BCL7A, MARK1, SCRN1, and NKX3.1 were methylated and their expression was upregulated by a demethylating agent. Their methylation frequencies in EBVaGC were significantly higher in comparison to EBV-negative gastric cancer [18].

The mechanism that induces cellular DNA methylation by EBV infection of the gastric epithelium is not fully understood. LMP2A can activate the transcription of DNA methyltransferase 1 (DNMT1) through the phosphorylation of STAT3 [37]. However, LMP2A is not expressed in all cases of EBVaGC [24], and EBVaGC patients are usually negative for LMP2A antibodies [38]. LMP1 can also induce aberrant DNA methylation by activating DNMT1 through the JNK signaling pathway [39] and by inducing DNA methylation in the host cells [40]. However, LMP1 is scarcely expressed, and the LMP1 protein is usually not expressed in EBVaGC [41]. Matsusaka et al. showed that when EBV was introduced into the EBV-negative/low-methylation epigenotype gastric cancer cell MKN7, DNA methylation was induced in the cells [42]. They recently described the observation of similar DNA methylation in non-neoplastic gastric epithelial cell line GES1 after EBV infection [43]. They concluded that DNA methylation could be attributed to EBV infection.

**Figure 2:**
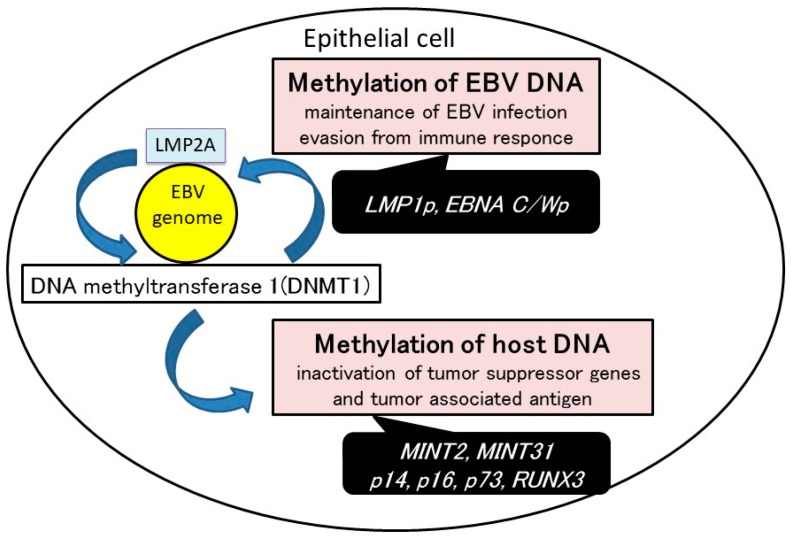
The mechanism of EBV-induced gastric carcinogenesis. DNA methylation can be induced by EBV infection in epithelial cells. Methylation of both viral and host DNA is one of the major mechanisms involved in the development of EBV-associated gastric carcinoma (EBVaGC). Viral DNA methylation regulates EBV latency type and inhibits the expression of EBV latent genes that are possible targets of cytotoxic T lymphocytes. Methylation of host cell DNA might lead to the progression of EBVaGC. The inactivation of cellular genes through DNA methylation might contribute to cell-cycle dysregulation and anti-apoptotic effects in EBVaGC.

Methylation of both viral and host DNA is one of the major mechanisms involved in the development of EBVaGC. DNA methylation could be induced by EBV infection in epithelial cells. Viral DNA methylation inhibits the expression of EBV latent genes. Methylation of host cell DNA-inactivated tumor suppressor genes and tumor associated antigens (Figure 2).

## 4. MicroRNA (miRNA)

miRNA consists of 21–25-bp non-coding RNA and is generated from primary miRNAs (pri-miRNAs) through a multi-step process. In the nucleus, pri-miRNAs are transcribed by RNA polymerase II as incomplete double-stranded RNAs and cleaved by the Drosha-DGCR8 complex to generate intermediate miRNA (precursor miRNA [pre-miRNA]). Pre-miRNAs are transported from the nucleus to the cytoplasm by Exportin5-RanGTP8 complex and are processed by the Dicer-TRBP2 complex to create mature miRNAs. miRNAs are captured by RNA-induced silencing complex (RISC). RISC binds to the 3’UTR of mRNA; RISC-miRNA suppresses translation and induces deadenylation to enhance mRNA degradation [44]. A total of 2588 miRNAs have been identified in *Homo sapiens*, and it is estimated that 30–50% of genes are regulated by miRNAs [45].

### 4.1. EBV Encoded miRNAs

**Figure 3:**
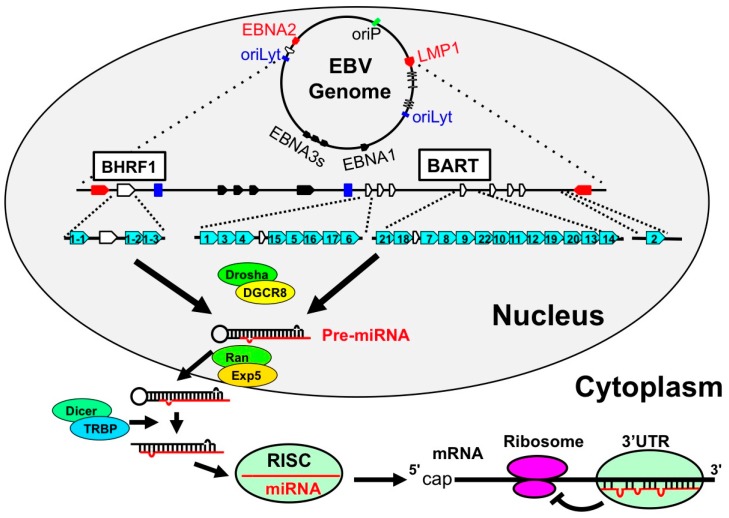
The genomic location of EBV-encoded miRNAs. In latently-infected cells, the EBV genome exists in the nucleus as a circular episome. The starting sites of viral replication are indicated as a green box (origin of plasmid replication; oriP) and blue boxes (origins of lytic replication; oriLyt). The coding regions of the latent membrane protein 1 (LMP1) and EBNA2 are indicated as red arrows. These are transcribed in antisense and sense orientations, respectively. EBNA3s and EBNA1 are indicated as black arrows. With regard to the BHRF miRNAs, BHRF1 ORF is encompassed with one 5′-end and two 3′-end miRNAs (light blue arrows). BART miRNAs are located in the intron region of noncoding RNA BART and are indicated as blue arrows. The white arrows indicate the BHRF and BART exons. (1) Drosha processing: pri-miRNAs are processed by Drosha-DGCR8 complex to become pre-miRNAs. (2) Pre-miRNA transport: pre-miRNAs are then transferred from the nucleus to the cytoplasm by Exportin5-RanGTP8 complex. (3) Dicer processing: pre-miRNAs are digested by Dicer-TRBP complex and miRNAs are generated. (4) RISC loading and translational inhibition: and miRNAs are incorporated into RNA-induced silencing complex (RISC) and specifically inhibit the translation of the targeted mRNA.

In 2004, Pfeffer et al. reported that EBV B95-8 strain has pri-miRNA-like structures and that viral miRNAs are produced using host enzymes [46]. Thereafter, it was found that many viral miRNAs were present in the BART intron region, which was lacking in the B95-8 strain. It has since been confirmed that EBV has 44 miRNAs (miR-BHRF1s, miR-BARTs: Table 2 and Figure 3) [47]. miR-BARTs are highly expressed in NPC and EBVaGC in comparison to EBV-positive B lymphoma [48]. In contrast, miR-BHRF1s are expressed in LCL, but not in Burkitt lymphoma, NPC, or EBVaGC [48]. EBV is expressed in a limited number of genes in EBVaGC. Thus, it was important to clarify the function of miR-BARTs in EBVaGC. miR-BART20-5p was found to target apoptosis-inducing factor, Bcl2-associated agonist of cell death (*BAD*) and induce the proliferation of gastric cancer cells by repressing the expression of BAD [49]. Moreover, miR-BART4-5p was found to target BH3 interacting domain death agonist *(BID*), which is a Bcl-2 family gene, and suppress apoptosis in gastric cancer cells [50]. In addition to targeting *BAD* and *BID*, miR-BARTs were shown to suppress cell death by targeting various apoptosis-related genes [51].

The mRNA profiles of epithelial cells and B cells are different, and it is hypothesized that the target genes of miR-BARTs also depend on the cell type. Kanda et al. reported that multiple miR-BARTs, including miR-BART22, targeted the epithelial cell differentiation marker NDRG1 [52]. This is an essential gene for maintaining epithelial cell differentiation. The effect of miR-BARTs is not limited to EBV-infected cells because miRNAs are secreted extracellularly by exosomes [21]. miR-BART11 is reported to cause the dedifferentiation of epithelial cells and tumor-infiltrating macrophages by targeting the cell differentiation-inducing factor *Forkhead Box P1* (*FOXP1*) [53].

In cases of EBVaGC involving latent EBV infection, the viral antigens are expressed at very low levels, allowing the virus to escape the immune system and a state of persistent infection to be maintained. miR-BART6 was shown to induce latent EBV infection [54]. In addition, miR-BART20-5p was shown to target lytic infection, inducing factors BZLF1 and BRLF1, and to induce latent viral infection [55]. Although the expression of BZLF1 was shown to be enhanced in a miR-BART-deleted EBV strain, it was found that the strain progressed to a lytic condition more easily in comparison to a wild-type strain [56].

BART miRNAs are more highly expressed in epithelial tumors than in B lymphoma [57]. BART transcription is regulated by its promoter regions, and P1 and P2 have been reported as BART promoters [58]. C/EBPα, β, and δ have been shown to interact with P2, and the expression of the C/EBPβ protein was also detected in epithelial tumors but not in B lymphoma [58]. Moreover, the P2 region was shown to be highly methylated in type I latency B lymphoma in comparison to type III latency B lymphoma, and treatment with decitabine (5-Aza-2′-deoxycytidine; DAC) led to the recovery of the P2 promoter activity with the high expression of BART miRNA [59]. BART promoters may regulate BART transcription through a cell-type-specific transcription factor and epigenetic modification.

These reports indicate that miR-BARTs have various roles, including the repression of apoptosis, the induction of dedifferentiation, and the maintenance of latent infection.

### 4.2. Host miRNAs

EBV derived microRNAs repress translation from viral and host mRNAs. Shinozaki et al. reported that EBV encoded EBNA1 and that LMP2A repressed the expression of pri-mir-200, which is a repressor of epithelial mesenchymal transition (EMT) [60]. In addition, Marquitz et al. analyzed the miRNA profile of AGS-EBV, an EBV experimentally-infected gastric carcinoma cell line, and reported that EBV infection reduced host expression levels of miR-200, miR-143-3p, and miR-146b [61]. The low expression of host miRNAs was reported to be associated with the prognosis of cancer [62]. Dicer is an essential factor in the generation of miRNAs, such as miR-200, and the low expression of Dicer enhances tumor metastasis via the induction of EMT [63]. EBV has been shown to encode miR-BART6 and to target Dicer [54]. These reports suggest that EBV infection changes the host miRNA profile and changes in this profile may enhance the metastatic activity of EBV-infected tumor cells.

## 5. Long Non-Coding RNA (lncRNA)

**Figure 4:**
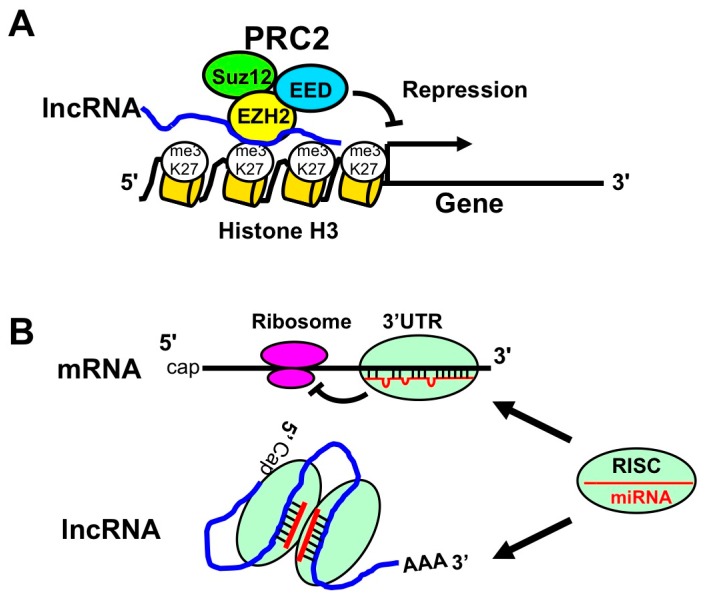
The role of long non-coding RNAs (lncRNAs). (**A**) LncRNA-PRC2 complex suppresses gene transcription. LncRNA binds to polycomb proteins, such as EZH2, EED, and Suz12. These proteins are recognized as PRC2. PRC2-lncRNA complex interacts with K27 of histone H3 and induces the trimethylation of K27 (me3K27). The resulting histone modification represses the transcription of targeted genes; (**B**) LncRNAs repress miRNA function by absorption of miRNA. The upper panel illustrates the well-known repression of translation by RISC-miRNA complex through its binding to 3′UTR of mRNA. The lower panel illustrates the interaction of lncRNA with miRNA via the targeting sequences. Since lncRNA absorbs miRNA, the association of miRNA with protein coding mRNAs will be decreased to increase the protein expression levels.

LncRNA is a long non-coding RNA of ≥200 bases, and 27,919 lncRNAs have been identified in humans [64]. The function of lncRNA is largely unknown. However, lncRNAs bind to polycomb complex (PRC1 and PRC2) and inhibit transcription via histone modification (Figure 4A). Moreover, lncRNAs also interact with miRNAs (instead of protein-coding mRNAs) and enhance translation (Figure 4B) [65].

### 5.1. EBV Encoded lncRNAs

EBV encodes viral miRNAs in the intron region of BART lncRNA [22]. However, BART lncRNA also regulates host mRNA expression. Recently, Marquitz et al. reported that BART lncRNA reduced the expression of amino acid transporter xCT, cadherin superfamily CDH11, and E3 ubiquitin ligase RNF144B [66]. xCT is a glutamate-cystine transporter that is highly expressed in cancer stem cells to erase reactive oxygen species [67]. Moreover, RNF144B, which is one of the ΔNp63 downstream genes, enhances the degradation of p21 to induce cell proliferation [68]. These reports suggest that BART lncRNA may repress tumor malignancy in EBVaGC. It has also been reported that several lncRNAs expressed by oriP latently infected cells with EBV [69]. The role of BART lncRNA and these viral lncRNAs in tumor malignancy remains unclear.

### 5.2. Host lncRNAs

EBV infection alters the mRNA expression profile, including the expression of lncRNAs, in tumor cells. RNU12, H19, RP11-359D14.3, SNHG8, and MIR143HG were shown to be highly expressed in EBVaGC [70]. The expression of H19 induces the proliferation of gastric carcinoma cells [71]. Moreover, H19 is expressed not only in EBVaGC, but also in lymphoblastoma, suggesting that H19 may be associated with EBV-related tumors [72].

These reports indicate that EBV induces tumor malignancy by regulating the expression of non-coding RNA in gastric carcinoma. Recently, several review articles reported on miRNA and lncRNA in relation to EBV and gastric cancer [73,74,75]. For example, it was introduced that HOTAIR, GAS5, and MEG3 were highly expressed in gastric carcinoma [75]. However, it is unclear that the role of these lncRNAs in EBVaGC. To understand the role of non-coding RNA in EBVaGC, further studies are needed.

## 6. Demethylating Agents in EBV-Associated Gastric Cancer

DAC and azacitidine are DNA demethylating agents that have shown clinical efficacy in the treatment of hematological malignancies [76,77,78]. DAC prolongs the overall survival of patients with myelodysplastic syndrome and acute myeloid leukemia. Combination therapy with anti-cancer agents and DAC also improves the prognosis of acute lymphocytic leukemia [79,80].

With regard to the clinical application of demethylating agents against EBVaGC, we investigated the effects of DAC against EBVaGC cell lines. DAC was found to induce G2/M arrest, apoptosis, and the expression of E-cadherin in SNU719 cells. The promoter regions of p73 and RUNX3 were demethylated, and their expression was upregulated by DAC treatment [81]. We also examined the effect of another demethylating agent, zebularine, and found that it inhibited the growth of SNU719 and NCC24 cells, which were derived from EBV-positive gastric adenocarcinoma [81].

It is known that DAC changes the expression of EBV genes that switch latent EBV infection to lytic EBV infection in infected cells [82,83,84,85]. BZLF1, a transcriptional activator that mediates the switch between the latent and the lytic forms of EBV infection, was found to be upregulated in SNU719 cells. The expression of BZLF1 causes apoptosis of the host cell and the release of EBV particles [86,87,88,89]. This upregulation of BZLF1 is considered to be one of the mechanisms underlying the anti-tumor effect of DAC in EBV-associated cancers. These facts strongly support the possible application of demethylating agents in the medical treatment of EBVaGC.

The efficacy of DAC in the treatment of solid tumors has not been reported [90]. Recently, a phase I study of 5-azacitidine prior to standard neoadjuvant chemotherapy was conducted for patients with gastric adenocarcinoma. Epigenetic priming with azacitidine plus chemotherapy was well-tolerated, and a multi-institutional phase II study is being developed using the established dose of azacytidine. Demethylating agents can be applied to the treatment of EBVaGC because promoter hypermethylation of the tumor suppressor gene is a key mechanism in the development of EBVaGC [22,91].

## 7. Conclusions

Methylation of both viral and host DNA is one of the major mechanisms involved in the development of EBVaGC. DNA methylation regulates the expression of miRNAs and lncRNAs coded in both viral and host DNA and is also involved in EBV-induced transformation of gastric cells. Epigenetic therapy could potentially be applied to the treatment of EBVaGC.

## Figures and Tables

**Table 1:** The pattern of Epstein Barr virus (EBV) latent gene expression and promoter usage.

Type of Latency	Latent Gene Expression	Promoter Hypermethylation			EBV-Associated Diseases
		Cp/Wp	Qp	LMP1p	
Type I	EBNA1	+	−	+	Burkitt lymphoma; Gastric cancer
Type II	EBNA1, LMP1, LMP2	+	−	+/−	Nasopharyngeal cancer, Hodgikin disease, NK/T cell lymphoma
Type III	EBNA1–6, LMP1, LMP2	−	−	−	Immunosuppressive lymphoma

EBV, Epstein-Barr virus; EBNA, Epstein-Barr Virus nuclear antigen; LMP1, Latent membrane protein 1; Cp, C promoter; Wp, W promoter.

**Table 2:** The role of miRNAs in EBVaGC.

	miRNA	Target	Role of Targeted Gene	Reference
EBV miRNAs	miR-BART3	DICE1	Apoptosis	[51]
		FEM1B	Apoptosis	[51]
		CASZ1a	Apoptosis	[51]
	miR-BART4-5p	BID	Apoptosis	[50]
	miR-BART6-5p	DICER1	Lytic replication-related genes	[51,54]
		DICER1	Repressor of EMT	[51,60]
		OCT1	Apoptosis	[46,51]
	miR-BART8	ARID2	Tumor suppressor gene	[51]
	miR-BART11	FOXP1	Cellular differentiation factor	[53]
	miR-BART16	CREBBP	Apoptosis	[51]
		SH2B3	Apoptosis	[51]
		TOMM22	Mitochondrial transporter	[51]
	miR-BART20-5p	BAD	Apoptosis	[49]
		BZLF1/BRLF1	Lytic replication-related genes	[49]
	miR-BART22	PPP3R1	Apoptosis	[51]
		PAK2	Apoptosis	[51]
		TP53INP1	Apoptosis	[51]
		NDRG1	Cellular differentiation factor	[52]
Host miRNA (Reduction)	miR-200	ZEB1, ZEB2	Repressor of EMT	[61,62]
	miR-143-3p	KLF4, ELK1	Stem cell factor	[61,62]
	miR-146b	STAT3	Inflammation (IL-6)	[61,62]

EBVaGC: Epstein-Barr virus-associated gastric carcinoma; EMT: epithelial mesenchymal transition.
